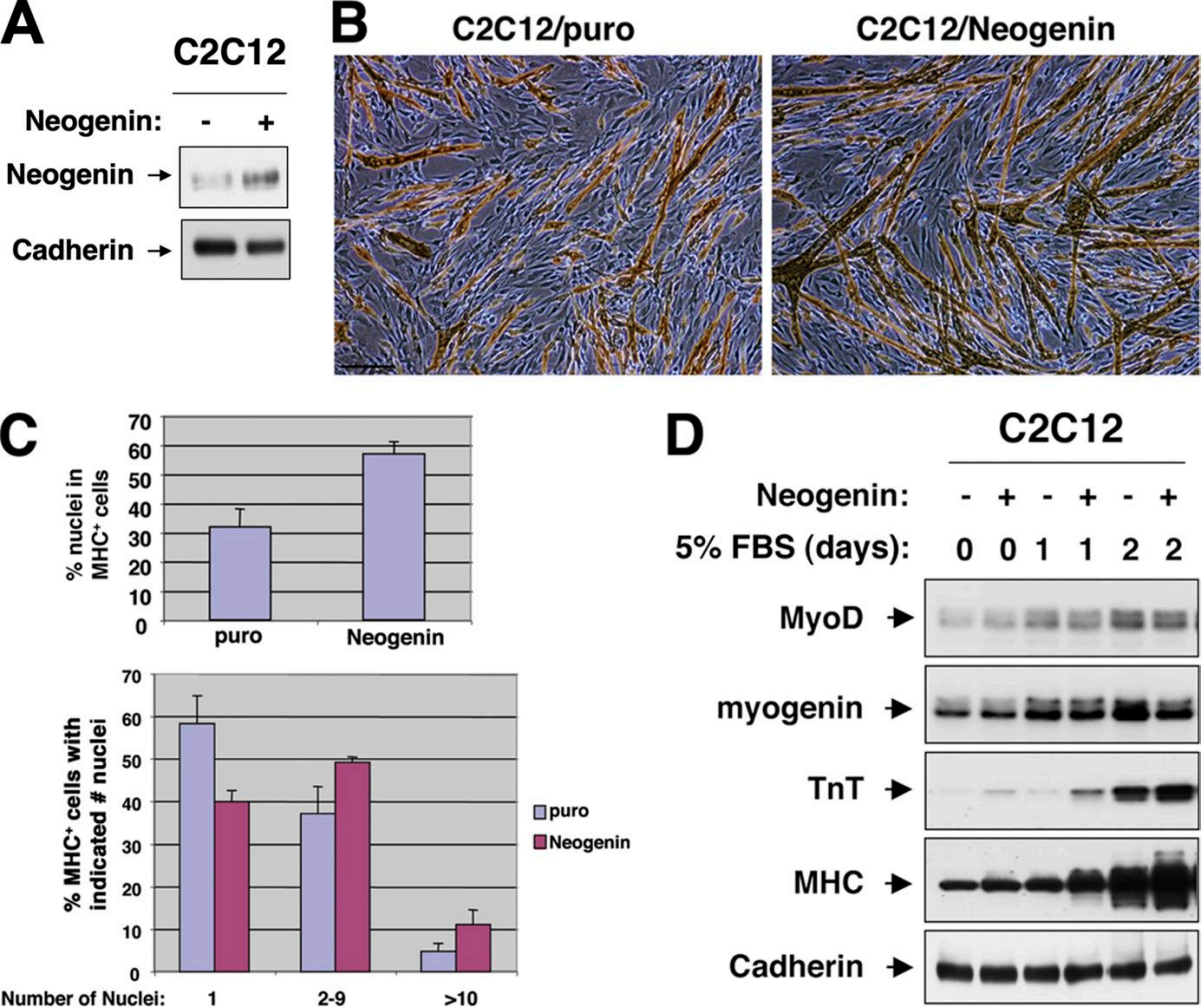# Correction: Netrins and neogenin promote myotube formation

**DOI:** 10.1083/jcb.20040503909082023c

**Published:** 2023-09-13

**Authors:** Jong-Sun Kang, Min-Jeong Yi, Wei Zhang, Jessica L. Feinleib, Francesca Cole, Robert S. Krauss

Vol. 167, No. 3 | https://doi.org/10.1083/jcb.200405039 | November 1, 2004

It was recently discovered that the Cadherin loading control for the Western blot in Fig. 4 C had been duplicated in Fig. 2 A. In checking the blots and records from that time, the authors found that the loading controls for both experiments had been imaged at the same time and on the same film, leading to the inadvertent selection of the wrong image for Fig. 2 A.

The corrected Fig. 2 is shown here and has been replaced in the paper. The conclusions of the paper are not affected by this error.

The error appears in print and in PDFs downloaded on or before September 8, 2023. The authors apologize for the error and any confusion.

**Figure 2:**